# American Dermatological Association

**Published:** 1885-10

**Authors:** 


### THE AMERICAN DERMATOLOGICAL ASSOCIATION.

The ninth annual meeting of the American Dermatological Association was held at Greenwich, Conn., August 26th, 27th, and 28th, 1885. The attendance was the largest recorded in the history of the Association.

After the usual address by the President, Dr. W. A. Hardaway, of St. Louis, the first paper was read by Dr. J. E. Graham, of Canada, in which a full description was given of an unusual case of tuberculo-ulcerative syphiloderm of hereditary origin. The paper was illustrated by colored drawings of the disease, which largely affected the arm and shoulder of a girl twenty years of age.

Dr. F. B. Greenough, of Boston, then read a paper entitled "Clinical Notes on Psoriasis," being a careful collation of the records of 394 cases of the disease treated in dispensary practice, illustrating clearly the more interesting features of the disease in their clinical preponderance. In the discussion which followed, it was shown with clearness that too much stress has been laid upon the disposition of psoriasis to affect the region about the extensor faces of the extremities, the disease, in more cases than is commonly supposed, selecting other portions of the body for amplest development.

Dr. Samuel Sherwell, of New York, followed, in the afternoon session, with a paper entitled "Remarks on a Moot Point in the Etiology of Psoriasis," in which a careful collation of authorities had been made in the effort to show the views generally held as to constitutional origin of the disorder.

Two papers, one by Dr. James C. White, of Boston, and the other by Dr. James Nevins Hyde, of Chicago, were read consecutively and discussed together, in the evening session of the first day. The former was entitled "The Treatment of Lupus by Parasiticides;" the latter, "On the Relations of Lupus Vulgaris to Tuberculosis," being based upon a study of twenty consecutive cases. Both authors held to a belief in the relationship of lupus to tuberculosis, as explained by the bacilli recognized and cultivated in the two disorders. Dr. White's report of the results of treatment by local applications of the mercuric bichloride in solution and ointment, was in a high degree satisfactory.

Dr. White, on the evening previous, had also read a comprehensive description of cases of Angioma Pigmentosum et atrophicum, observed by himself, and illustrated by admirable photographs. These, with the cases previously reported by R. W. Taylor and L. A. Duhring, are the only instances of the disorder thus far reported as observed in America.

Dr. A. R. Robinson, of New York, read, on the morning of the second day, a paper entitled "On the Histology of the Vegetable Parasites," more particularly relating to the anatomical portions of the hair follicle invaded by the trichophyton. The paper was the result of the careful microscopical observations of the author, for which he has a deserved reputation.

His paper, as well as that of Dr. Heitzmann, of New York, which followed, " On the Structure of the Derma, and of the elastic tissue in it," was illustrated by microscopical demonstrations.

The paper by Dr. W. A. Hardaway, of St. Louis, on " Myoma of the Skin," was of interest as showing the pathological character of many of the tumors which from clinical symptoms might be assumed to to be purely neuromatous. The paper was a valuable contribution to the study of both neuroma and myoma of the skin.

Dr. L. A. Duhring, of Philadelphia, in the afternoon, followed with a paper on the " Relation of Herpes Gestationis and certain other forms of Disease to Dermatitis Herpetiformis." The author, in this contribution, merely pushed further the work which he has been exhaustively conducting in the yet poorly-defined field of dermatitis herpetiformis, which has been during the last year illustrated by several papers from his pen.

Dr. G. H. Tilden, of Boston, followed with a paper on Mycosis Fongöide, based upon a case under his observation. The paper was an exhaustive contribution to the subject, the author taking the ground not held by the majority of those present, viz: that the disease under description was not purely sarcomatous in character.

Dr. R. B. Morison, of Baltimore, reported an unusual case of Tylosis of the hands, illustrated by a sketch of those members in a negro who was a victim of the disorder. Opinions were divided as to the nature of this malady, whether it was a variety of the " *Mal Pérforant du Pied*," or was purely a tylosis in the case of a person whose hands were excessively exposed to injurious influences in the labor of shoveling coal.

Dr. L. N. Denslow, of Minnesota, read a paper on Urethral Irritation as a source of certain neuroses, with special reference to acne; and followed this on the third day with a second short paper reporting a case of Syphilitic Aphasia and Paraple7ia, followed by death, with an account of the autopsy. Both papers were of interest, the former being apparently confirmatory of the views expressed by Dr. Sherwell in his paper on a similar subject a year before.

On the morning of the third day Dr. C. Heitzmann, in his remarks on "Electrolysis and other Practical Topics," gave the signal for a full discussion of the several methods of removing hairs, port-wine marks, and small growths, by the continued electric current. The author in his paper discussed, also the treatment of freckles and, of seborrhœa of the scalp.

Dr. H. W. Stelwagon, of Philadelphia, read a paper on the employment of the Oleates in Skin Diseases, which elicited an interesting discussion. Both the author and those who heard him were in agreement as to the valuelessness of most of the oleates lauded for treatment of cutaneous maladies.

Dr. R. W. Taylor's paper on Syphilitic Reinfection was a contribution of three more cases to those previously reported in literature. They were all, from the first, observed by himself, and reported with the detail and minuteness requisite for establishing the essential fact of both a first and second syphilitic infection.

Dr. Hyde, chairman of the committee on statistics, reported 9,346 cases of cutaneous disease registered during the year by members, arranged in the tables and according to the nomenclature of the Association.

Adjourned to meet at the same place, in the same week of August of the ensuing year.

THE WESTERN SOCIETY OF PSYCHICAL RESEARCH.

This society was organized in May of the current year. It has already held several meetings, elected officers and members, and outlined its work. The president, Professor A. R. Jackson, at the meeting held on June 30th, briefly stated the objects of the society to be the investigation of the phenomena of so-called apparitions, mesmerism, mind-reading, clairvoyance, spiritualistic manifestations, etc., and, in the language of the English Society for Psychical Research, "to approach these various problems without prejudice or prepossession of any kind, and in the same spirit of exact and unimpassioned inquiry which has enabled science to solve so many problems once not less obscure nor less hotly debated."

The western society has been founded for the purpose of aiding in the work so successfully commenced by the societies in England and Boston. Five committees have been organized, as follows:

1. Committee on Thought-Transference. Rev. H. W. Thomas, Chairman.

2. Committee on Hypnotism, Clairvoyance and Somnambulism. Dr. E. J. Kuh, Chairman.

3. Committee on Apparitions and Haunted Houses. Professor Rodney Welch, Chairman.

4. Committee on Physical Phenomena. D. W. Chapman, Chairman.

5. Committee on Psychopathy — mind and faith cure, etc. Col. A. N. Waterman, Chairman.

At the last meeting the secretary reported a membership of over one hundred. The amount of valuable work done, and the scientific accuracy of the reports made by the English society, have doubtless furnished the motive for the present organization.

